# Supplementary material for: Social isolation in mid-life: associations with psychological distress, life satisfaction and self-rated health in two successive British birth cohorts
Source: Soc Psychiatry Psychiatr Epidemiol. 2026 Jan 19;61(5):885–96. doi: 10.1007/s00127-025-03041-9 (PMC13156202; doi:10.1007/s00127-025-03041-9)
Supplement: Supplementary file 1 — Supplementary Material 1 [file 127_2025_3041_MOESM1_ESM.pdf]

Supplementary File S1.

Social isolation in mid-life: associations with psychological distress, life satisfaction and self-rated health in two successive British birth cohorts

Rosie Mansfield [a*] Marcus Richards [b], George B. Ploubidis [a], Morag Henderson [a+], Praveetha Patalay [a, b+]

[a] Centre for Longitudinal Studies, University College London, London, United Kingdom

[b] MRC Unit for Lifelong Health and Ageing, University College London, London, United Kingdom

*Corresponding author: Professor Praveetha Patalay, p.patalay@ucl.ac.uk

+Shared senior authors

Table of Contents

Table S1. *Descriptive statistics for demographic, socioeconomic and health covariates, social isolation indicators and mental health outcomes using complete case data* 3

Table S2. *Descriptive statistics for demographic, socioeconomic and health covariates using multiply imputed data m=50 (1970 BCS N = 16,585 and 1958 NCDS = 15,806)* 5

Table S3. *Harmonisation of demographic, socioeconomic and health covariates, social isolation indicators and mental health outcomes* 6

Table S4. *N(%) of missing cases for covariates, social isolation indicators and mental health outcomes (Full sample N = 32,391, 1970 BCS N = 16,585, 1958 NCDS N = 15,806)* 16

Missing data strategy 18

Table S5. *Linear multivariable regression models for each separate social isolation indicator to identify (1) independent effects of each social isolation indicator and (2) cohort effects in these independent effects on psychological distress, life satisfaction and self-rated general health using multiply imputed data m=50 (Full sample N = 32,391, Males N = 15,810, Females N = 15,221)* 19

Table S6. *Linear multivariable regression models for each separate social isolation indicator to identify (1) independent effects of each social isolation indicator and (2) cohort effects in these independent effects on psychological distress, life satisfaction and self-rated general health – complete case* 22

Table S7. *Linear multivariable regression models for each separate social isolation indicator to identify (1) independent effects of each social isolation indicator and (2) cohort effects in these independent effects on psychological distress, life satisfaction and self-rated general health using impute and delete data* 24

Figure S1 (a, b, c and d). *Model predicted marginal mean plots for significant (p<.10) social isolation by cohort interactions on mental health outcomes* 26

Figure S2. *Model predicted marginal mean plot for cumulative social isolation score by cohort interaction on self-rated general health* 27

Table S8. *Linear multivariable regression models for the cumulative social isolation score to identify 1) 'dose response' effects using the cumulative social isolation score, and 2) cohort effects in any 'dose-response' associations on psychological distress, life satisfaction and self-rated general health – complete case and impute and delete models* 28

Table S9. *Linear multivariable regression models for the interaction between multiple social isolation indicators to identify 1) the additive and multiplicative interaction effects, and 2) cohort effects in any interaction associations on psychological distress, life satisfaction and self-rated general health using multiply imputed data m=50 (Full sample N = 32,391, Males N = 15,810, Females N = 15,221)* 30

Table S10. *Linear multivariable regression models for the interaction between multiple social isolation indicators to identify 1) the additive and multiplicative interaction effects, and 2) cohort effects in any interaction associations on psychological distress, life satisfaction and self-rated general health using complete case data* 32

Table S11. *Linear multivariable regression models for the interaction between multiple social isolation indicators to identify 1) the additive and multiplicative interaction effects, and 2) cohort effects in any interaction associations on psychological distress, life satisfaction and self-rated general health using impute and delete data* 35

Latent Class Analysis Methodology 37

Table S12. *Results from the latent class analyses (1-5 classes) including goodness-of-fit statistics (full sample with 1970 BCS and 1958 cohorts combined N=19,902)* 37

Table S13. *Class membership (N(%)) for full sample and by sex and cohort* 38

Table S1. *Descriptive statistics for demographic, socioeconomic and health covariates, social isolation indicators and mental health outcomes using complete case data*

	1970 BCS			1958 NCDS		
Variable	N	Age(year)	%	N	Age(year)	%
Demographic, Socioeconomic and Health Covariates						
Sex (if female)	15,226	Birth	7,457(48.98)	15,805	Birth	7,764(49.12)
Self-reported sex (if female)	8,804	38 (2008)	4,638(52.68)	11,213	42 (1999)	5,712(50.94)
Education – highest qualification - educated to degree level (if no degree)	8,764	38 (2008)	8,418(96.05)	15,805	42 (1999)	15,325(96.96)
Socioeconomic status – self-reported financial difficulties (if yes)	8,773	38 (2008)	797(9.08)	11,174	42 (1999)	896(8.02)
Socioeconomic status – occupational social class	7,423	38 (2008)		9,487	42 (1999)	
Professional/Managerial-technical			3,739(50.37)			4,059(42.78)
Intermediate			2,771(37.33)			3,937(41.50)
Partly skilled/Unskilled			881(11.87)			1,471(15.51)
Others			32(0.43)			20(0.21)
Socioeconomic status – homeownership (if no)	8,757	38 (2008)	1,922(21.95)	11,131	42 (1999)	2,091(18.79)
Health – self-rated general health (Mean (SE))	8,770	38 (2008)	3.73(1.01)	11,171	42 (1999)	3.09(0.76)
Health – limiting long standing illness (if yes)	8,803	38 (2008)	1,394(15.84)	11,171	42 (1999)	3,234(28.95)
Health – psychological distress (Mean (SE))	9,445	34 (2004)	1.67(1.90)	11,076	42 (1999)	1.51(1.78)
Health – life satisfaction (Mean (SE))	9,441	34 (2004)	7.41(1.80)	11,069	42 (1999)	7.30(1.91)
Social Isolation Indicators						
Living alone (if yes)	9,802	42 (2012)	949(9.68)	9,432	46 (2004)	870(9.22)
Lack of regular contact with friends and relatives outside of the household (if yes)	9,749	42 (2012)	345(3.54)	8,482	44 (2002)	79(0.93)
Out of employment (if yes)	9,767	42 (2012)	1,400(14.33)	9,448	46 (2004)	1,164(12.32)
Lack of community engagement (if yes)	6,512	42 (2012)<br>46 (2016)	4,869(74.77)	9,441	46 (2004)	7,259(76.89)

Total social isolation score (0-3)*	6,497	42 (2012)		7,816	46 (2004)	
0			1,307(20.12)			1,546(19.78)
1			4,062(62.52)			5,078(64.97)
2			1,022(15.73)			1,088(13.92)
3			106(1.63)			104(1.33)
Mental Health Outcomes						
Psychological distress (Mean(SD))	7,866	46 (2016)	1.77(2.13)	7,866	50 (2008)	1.77(2.13)
Subjective wellbeing - life satisfaction (Mean(SD))	8,487	46 (2016)	7.35(1.89)	9,631	50 (2008)	7.29(1.84)
Self-rated general health (Mean(SD))	8,576	46 (2016)	3.44(1.10)	9,733	50 (2008)	3.48(1.11)

Note: * Due to small counts for social isolation scores of four, scores of three and four are combined.

Table S2. *Descriptive statistics for demographic, socioeconomic and health covariates using multiply imputed data m=50 (1970 BCS N = 16,585 and 1958 NCDS = 15,806)*

	1970 BCS		1958 NCDS	
Variable	Age (year)	%	Age (year)	%
Self-reported sex (if female)	38 (2008)	53.17	42 (1999)	50.64
Education – highest qualification - educated to degree level (if no degree)	38 (2008)	96.18	42 (1999)	96.96
Socioeconomic status – self-reported financial difficulties (if yes)	38 (2008)	10.13	42 (1999)	8.40
Socioeconomic status – occupational social class	38 (2008)		42 (1999)	
Professional/Managerial-technical		45.96		38.31
Intermediate		38.88		42.44
Partly skilled/Unskilled		14.51		18.92
Others		0.66		0.33
Socioeconomic status – homeownership (if no)	38 (2008)	24.65	42 (1999)	20.10
Health – self-rated general health (Mean(SE))	38 (2008)	3.67(.01)	42 (1999)	3.07(.01)
Health – limiting long standing illness (if yes)	38 (2008)	17.18	42 (1999)	29.81
Health – psychological distress (Mean(SE))	34 (2004)	1.75(.02)	42 (1999)	1.55(.02)
Health – life satisfaction (Mean(SE))	34 (2004)	7.33(.02)	42 (1999)	7.27(.02)

Table S3. *Harmonisation of demographic, socioeconomic and health covariates, social isolation indicators and mental health outcomes*

Measure	Cohort	Age (year)	Variable	Item/Questionnaire	Recode
Demographic, Socioeconomic and Health Covariates					
Sex	1970 BCS	38 (2008)	b8cmsex	Self-reported cohort member's sex	Males = 0 Females = 1 recode 1=0 2=1
	1958 NCDS	42 (1999)	n622_6	Self-reported cohort member's sex	Males = 0 Females = 1 recode 1=0 2=1
Education – highest qualification – educated to degree level	1970 BCS	38 (2008)	bd8achq1	Highest Academic Qualification CM obtained in 2008 survey<br>-1 Level not calculable<br>0 No academic qualification<br>1 GCSE D-E<br>2 Other Scottish Quals<br>3 GCSE A-C, Scot Standards/Intermediate 1<br>4 Scot Intermediate 2 / AS Levels or 1 A Level<br>5 2+ A Levels, Scot Higher/6th<br>6 Diploma<br>7 Degree, PGCE, Other Degree qual<br>8 Higher Degree	Recode Degree, PGCE, Other Degree qual<br>8 Higher Degree = 0 anything else = 0 and no degree = 1
	1958 NCDS	42 (1999)	numdeg	No. of degrees CM has obtained?	Recode any degree = 0 no degree = 1

Socioeconomic status – self-reported financial difficulties	1970 BCS	38 (2008)	b8finnow	How well would you say you personally are managing financially these days. Would you say you are<br>1 ...living comfortably<br>2 doing all right<br>3 just about getting by<br>4 finding it quite difficult or<br>5 finding it very difficult?	Recode 1 living comfortably 2 doing all right and 3 just about getting by = 0 and 4 finding it quite difficult and 5 finding it very difficult = 1
	1958 NCDS	42 (1999)	finnow	How well would you say you personally are managing financially these days. Would you say you are<br>1 ...living comfortably<br>2 doing all right<br>3 just about getting by<br>4 finding it quite difficult or<br>5 finding it very difficult?	Recode 1 living comfortably 2 doing all right and 3 just about getting by = 0 and 4 finding it quite difficult and 5 finding it very difficult = 1
Socioeconomic status – occupational social class	1970 BCS	38 (2008)	b8sc	(Current Job) Social Class<br>1 I Professional<br>2 II Managerial-technical<br>3.1<br>3.2<br>4 IV Partly skilled<br>5 Unskilled<br>6 Others	Recode<br>I Professional<br>II Managerial-technical = 0<br>3.1/3.2 = 1<br>IV Partly skilled<br>Unskilled = 2<br>Others = 3
	1958 NCDS	42 (1999)	sc	(Current Job) Social Class<br>1 I Professional<br>2 II Managerial-technical	Recode<br>I Professional

				3.1<br>3.2<br>4 IV Partly skilled<br>5 Unskilled<br>6 Others	II Managerial-technical = 0<br>3.1/3.2 = 1<br>IV Partly skilled<br>Unskilled = 2<br>Others = 3
Socioeconomic status – home ownership	1970 BCS	38 (2008)	b8ten2	Do you own or rent your home or have some other arrangement?<br>1 Own - outright<br>2 Own - buying with help of a mortgage<br>3 Pay part rent and part mortgage<br>4 Rent it<br>5 Live here rent-free<br>6 Squatting<br>7 Other	Recode<br>1 Own - outright<br>2 Own - buying with help of a mortgage<br>3 Pay part rent and part mortgage = 0<br>4 Rent it<br>5 Live here rent-free<br>6 Squatting<br>7 Other = 1
	1958 NCDS	42 (1999)	tenure2	Do you own or rent your home or have some other arrangement?<br>1 Own - outright<br>2 Own - buying with help of a mortgage<br>3 Pay part rent and part mortgage<br>4 Rent it<br>5 Live here rent-free<br>6 Squatting<br>7 Other	Recode<br>1 Own - outright<br>2 Own - buying with help of a mortgage<br>3 Pay part rent and part mortgage = 0<br>4 Rent it<br>5 Live here rent-free<br>6 Squatting<br>7 Other = 1

Health – self-rated general health	1970 BCS	38 (2008)	b8hlthgn	In general, would you say your health is...<br>1 Excellent<br>2 Very good<br>3 Good<br>4 Fair<br>5 Poor	Recode so that high scores = better health 5 = “Excellent”, 1 = “Poor”
	1958 NCDS	42 (1999)	hlthgen	How would you describe your health generally? Would you say it is ...<br>1 Excellent<br>2 Good<br>3 Fair<br>4 Poor	Recode so that high scores = better health 4 = “Excellent”, 1 = “Poor”
Health – limiting long standing illness	1970 BCS	38 (2008)	b8lsiotr<br>b8khllt	Whether CM Has Any (Other) Long Standing Illness/ Disability<br>Whether Health Limits Everyday Activities	Combine items to get complete picture – if limiting long standing illness = 1
	1958 NCDS	42 (1999)	lsiany2	Do you have any long-standing illness, disability or infirmity? By long-standing I mean anything that has troubled you over a period of time, or that is likely to affect you over a period of time? Yes/no	Recode yes = 1 no = 0
Health – psychological distress	1970 BCS	34 (2004)	b7mal02<br>b7mal03<br>b7mal05<br>b7mal09<br>b7mal12<br>b7mal14<br>b7mal16	Malaise 9-item Questionnaire	Total score

			b7mal20<br>b7mal21		
	1958 NCDS	42 (1999)	mal02 mal03<br>mal05 mal09<br>mal12 mal14<br>mal16 mal20<br>mal21	Malaise 24-item Questionnaire reduced down to match the 9-item Questionnaire	Total score
Health – life satisfaction	1970 BCS	34 (2004)	b7lifet1	Here is a scale from 0 to 10, where '0' means that you are completely dissatisfied and '10' means that you are completely satisfied. Please enter the number which corresponds with how satisfied or dissatisfied you are with the way life has turned out so far	0-10
	1958 NCDS	42 (1999)	lifesat1	Here is a scale from 0-10 where '0' means that you are completely dissatisfied and '10' means that you are completely satisfied. Please enter the number which corresponds with how satisfied or dissatisfied you are about the way your life has turned out so far	0-10
Social Isolation Indicators					
Living alone	1970 BCS	42 (2012)	BD9HSIZE<br>B9GSLIVE<br>B9GRTOK	Derived Household Size (bcs70_2012_derived.dta)<br>HHgrid: whether person living with CM at interview (bcs70_2012_persongrid.dta)<br>HHgrid: Person's relationship to CM (bcs70_2012_persongrid.dta)	Derived variable for living alone where living alone = 1 using household grid and household member's relationship to cohort member –

					cross-checked against the study derived variable for household size
	1958 NCDS	46 (2004)	nd7numhh<br>n7rtok12-n7rtok20	(Derived) Total number of people in household<br>Person's relationship to Cohort Member	Derived variable for living alone where living alone = 1 using household grid and household member's relationship to cohort member – cross-checked against the study derived variable for household size
Lack of regular contact with friends and relatives outside of the household	1970 BCS	42 (2012)	B9FREMT<br>B9FAMMT	How often do you meet up with any of your friends? (bcs70_2012_flatfile.dta)<br>How often do you meet up with any members of your family who you do not live with? (bcs70_2012_flatfile.dta)	Recode to combine frequency of contact with family and friends and to indicate social isolation i.e., less than monthly contact = 1
	1958 NCDS	44 (2002)	seerels<br>visrels<br>numpals	How often do you have regular contact with relatives outside your household?	Recode to combine frequency of

				How often do you visit or are you visited by relatives who live outside your household? How often do you have regular contact with friends or acquaintances outside your household?	contact with family and friends and to indicate social isolation i.e., less than monthly contact = 1
Out of employment	1970 BCS	42 (2012)	BD9ECACT	(Derived) Current economic activity status	Recode so that being in neither education nor employment = 1
	1958 NCDS	46 (2004)	nd7ecact	(Derived) Current economic activity status	Recode so that being in neither education nor employment = 1
Lack of community engagement	1970 BCS	42 (2012)<br>46 (2016)	B10Q26<br>B10Q27<br>B9SCQ23<br>B9SCQ1W<br>B9SCQ1X	Whether CM is currently a member of any organisations, clubs or societies? How often CM participates in meetings/events/activities of any organisations?<br>How often, if ever, do you attend any kind of religious service or meeting?<br>How often have you done each of the following activities in the last 12 months? (voluntary organisation) (voluntary work)	Generate variables for membership to community group or organisation, volunteering and regular religious activity (i.e., at least monthly) then generate social isolation indicator for community context - isolated across 2/3 no group

					membership, no volunteering and no regular religious activity = 1
	1958 NCDS	46 (2004)	nd7hobby<br>nd7othg<br>n7rnoweq<br>nd7youth<br>nd7polit<br>nd7eco<br>nd7othv<br>nd7local	I'd like you to think about any groups, clubs or organisations that you've been involved with since we last saw you in [^date of last interview]<br>How often, if ever, do you attend any kind of religious service or meeting?	Generate variables for membership to community group or organisation, volunteering and regular religious activity (i.e., at least monthly) then generate social isolation indicator for community context - isolated across 2/3 no group membership, no volunteering and no regular religious activity = 1
Total social isolation score	1970 BCS	42 (2012)	Living alone<br><br>Lack of regular contact with	Sum of all social isolation indicators – capped at 3 i.e., those scoring 4 combined with those scoring 3 due to very low counts.	Sum of all social isolation indicators (0-3)
	1958 NCDS	46 (2004)			

			friends and relatives outside of the household<br><br>Out of employment<br><br>Lack of community engagement		
Mental Health Outcomes					
Psychological distress	1970 BCS	46 (2016)	B10Q28A<br>B10Q28B<br>B10Q28C<br>B10Q28D<br>B10Q28E<br>B10Q28F<br>B10Q28G<br>B10Q28H<br>B10Q28I	Malaise 9-item Questionnaire	Total score
	1958 NCDS	50 (2008)	N8MAL02<br>N8MAL03<br>N8MAL05<br>N8MAL09<br>N8MAL12<br>N8MAL14<br>N8MAL16<br>N8MAL20<br>N8MAL21	Malaise 9-item Questionnaire	Total score

Subjective wellbeing - life satisfaction	1970 BCS	46 (2016)	B10LIFST1	Here is a scale from 0 to 10, where '0' means that you are completely dissatisfied and '10' means that you are completely satisfied. Please enter the number which corresponds with how satisfied or dissatisfied you are with the way life has turned out so far	0-10
	1958 NCDS	50 (2008)	N8LIFET1	Here is a scale from 0 to 10, where '0' means that you are completely dissatisfied and '10' means that you are completely satisfied. Please enter the number which corresponds with how satisfied or dissatisfied you are with the way life has turned out so far	0-10
Self-rated general health	1970 BCS	46 (2016)	B10HLTHGN	In general, would you say your health is 1. excellent 2. very good 3. good 4. fair 5. poor?	Reverse code so that excellent health = 5
	1958 NCDS	50 (2008)	N8HLTHGN	In general, would you say your health is 1. excellent 2. very good 3. good 4. fair 5. poor?	Reverse code so that excellent health = 5

Table S4. *N(%) of missing cases for covariates, social isolation indicators and mental health outcomes (Full sample N = 32,391, 1970 BCS N = 16,585, 1958 NCDS N = 15,806)*

Measure	Cohort	Age (year)	N(%) missing	Study outcome non-response
Demographic, Socioeconomic and Health Covariates				
Sex (at birth)<br>Self-reported sex	1970 BCS	38 (2008)	1,359(8.19)<br>7,781(46.92)	-
	1958 NCDS	42 (1999)	1(0.01)<br>4,593(29.06)	-
Education – highest qualification - educated to degree level	1970 BCS	38 (2008)	7,821(47.16)	-
	1958 NCDS	42 (1999)	1(0.01)	-
Socioeconomic status – self-reported financial difficulties	1970 BCS	38 (2008)	7,812(47.10)	-
	1958 NCDS	42 (1999)	4,632(29.31)	-
Socioeconomic status – occupational social class	1970 BCS	38 (2008)	9,162(55.24)	-
	1958 NCDS	42 (1999)	6,319(39.98)	-
Socioeconomic status – home ownership	1970 BCS	38 (2008)	7,828(47.20)	-
	1958 NCDS	42 (1999)	4,675(29.58)	-
Health – self-rated general health	1970 BCS	38 (2008)	7,815(47.12)	-
	1958 NCDS	42 (1999)	4,635(29.32)	-
Health – limiting long standing illness	1970 BCS	38 (2008)	7,782(46.92)	-
	1958 NCDS	42 (1999)	4,635(29.32)	-
Health – psychological distress	1970 BCS	34 (2004)	7,140(43.05)	-
	1958 NCDS	42 (1999)	4,730(29.93)	-
Health – life satisfaction	1970 BCS	34 (2004)	7,144(43.08)	-
	1958 NCDS	42 (1999)	4,737(29.97)	

Social Isolation Indicators				
Living alone	1970 BCS	42 (2012)	6,783(40.90)	-
	1958 NCDS	46 (2004)	6,374(40.33)	-
Lack of regular contact with friends and relatives outside of the household	1970 BCS	42 (2012)	6,836(41.22)	-
	1958 NCDS	44 (2002)	7,324(46.34)	-
Out of employment	1970 BCS	42 (2012)	6,818(41.11)	-
	1958 NCDS	46 (2004)	6,358(40.23)	-
Lack of community engagement	1970 BCS	42 (2012)<br>46 (2016)	10,073(60.74)	-
	1958 NCDS	46 (2004)	6,365(40.27)	-
Total social isolation score (0-3)*	1970 BCS	42 (2012)	10,088(60.83)	-
	1958 NCDS	46 (2004)	7,990(50.55)	-
Health Outcomes				
Psychological distress	1970 BCS	46 (2016)	8,719(52.57)	715(4.31%)
	1958 NCDS	50 (2008)	6,172(39.05)	156(0.99%)
Subjective wellbeing - life satisfaction	1970 BCS	46 (2016)	8,098(48.83)	94(0.57%)
	1958 NCDS	50 (2008)	6,175(39.07)	159(1.01%)
Self-rated general health	1970 BCS	46 (2016)	8,009(48.29)	5(0.03%)
	1958 NCDS	50 (2008)	6,073(38.42)	57(0.36%)

Note: *8,004(48.26%) of cases in 1970 BCS were unproductive in the most recent data collection sweep (age 46) i.e., they did not take part at all. Any missingness above and beyond 8,004 cases in the outcome is due to item non-response. 6,016(38.06%) unproductive cases in 1958 NCDS. * Due to small counts for social isolation scores of four, scores of three and four are combined.*

Missing data strategy

Non-randomness in discontinued participation and item non-response can lead to biases in estimates. Therefore, it was important to retain as much data as possible from underrepresented groups and account for non-response biases in analyses. Predictors of non-response in 1970 BCS and 1958 NCDS have been identified in previous research, improving the plausibility of the missing at random (MAR) assumption (Mostafa & Wiggins, 2014; Mostafa et al., 2021). The selection of auxiliary variables in the current study i.e., variables known to predict missingness which therefore help estimate imputed values, were informed by this earlier work (Mustillo & Kwon, 2015). The selected auxiliary variables (e.g., birth weight, parents' social class and education level, home ownership, and childhood cognitive ability) and all study variables were included in multiple imputation (MI) models using chained equations. Item non-response on study outcomes was low (<5%) however, due to lack of participation in the most recent data collection sweep, between 38-53% of cohort members were missing this information. MI is preferable to complete case analysis and produces less biased estimates, even with a relatively high percentage of missing data (Lee & Huber, 2021). Based on the overall proportions of missingness in the outcomes and standard recommendations, we chose to run 50 imputations (Sullivan et al., 2015; Lee & Huber, 2021) (see Supplementary Table S4. for more information on levels of missing data). For some auxiliary variables, response options were collapsed to reduce the number of categories to help imputation models converge. Due to working with two cohort studies, social isolation indicators were first recoded to ensure consistency across cohorts. Imputations were run using derived variables to ensure that the imputations and analyses were fully compatible. Sensitivity analyses were conducted for all models using an 'impute and delete' method (Von Hippel, 2007), analysing only those with data on study outcomes. This method can introduce biases in the presence of auxiliary variables associated with incomplete outcomes (Sullivan et al., 2015), it was therefore decided to include these models as supplementary as opposed to choosing this method for the main analyses. Results in a complete case sample are also presented as supplementary material.

Table S5. *Correlations between the social isolation and health outcome variables in each cohort*

	1970 BCS				1958 NCDS			
**Outcome variables**	Psychological distress	Life satisfaction	General Health		Psychological distress	Life satisfaction	General Health	
Psychological distress	1				1			
Life satisfaction	-0.47	1			-0.43	1		
General health	-0.45	0.38	1		-0.40	0.33	1	
**Social Isolation Indicators**	Living alone	Lack of regular contact with friends and relatives	Out of employment	Lack of community engagement	Living alone	Lack of regular contact with friends and relatives	Out of employment	Lack of community engagement
Living alone	1				1			
Lack of regular contact with friends and relatives	-0.01	1			-0.01	1		
Out of employment	0.07	0.03	1		0.07	0.01	1	
Lack of community engagement	0.04	0.04	-0.02	1	0.04	0.04	0.01	1

Table S6. *Linear multivariable regression models for each separate social isolation indicator to identify (1) independent effects of each social isolation indicator and (2) cohort effects in these independent effects on psychological distress, life satisfaction and self-rated general health using multiply imputed data m=50 (Full sample N = 32,391, Males N = 15,810, Females N = 15,221)*

All coefficients presented are for each independent social isolation indicator only (exposure)	Psychological distress coef [95% CI]		Life satisfaction coef [95% CI]		Self-rated general health coef [95% CI]	
	(1)Independent	(2)*Cohort	(1)Independent	(2)*Cohort	(1)Independent	(2)*Cohort
Living alone						
Full sample model	.029 [-.019 - .076]	.008 [-.081 - .097]	-.205*** [-.254 - -.155]	.069 [-.026 - .164]	-.040 [-.090 - .009]	-.139*** [-.231 - -.047]
Males only	.026 [-.031 - .083]	-.016 [-.124 - .091]	-.203*** [-.262 - -.144]	.082 [-.033 - .197]	-.053* [-.114 - .008]	-.133** [-.248 - -.018]
Females only	.020 [-.044 - .085]	.048 [-.078 - .174]	-.223*** [-.291 - -.155]	.052 [-.092 - .196]	-.038 [-.104 - .029]	-.137** [-.262 - -.012]
Lack of regular contact with friends and relatives outside of the household						
Full sample model	.066 [-.027 - .159]	-.190* [-.406 - .027]	-.206*** [-.306 - -.106]	.078 [-.145 - .301]	-.094** [-.182 - -.006]	-.062 [-.284 - .160]
Males only	.042 [-.074 - .158]	-.191 [-.449 - .068]	-.198*** [-.314 - -.083]	.051 [-.209 - .310]	-.091* [-.199 - .018]	-.058 [-.336 - .220]
Females only	.081 [-.043 - .205]	-.204 [-.523 - .116]	-.232*** [-.375 - -.088]	.098 [-.257 - .453]	-.095 [-.222 - .033]	-.081 [-.385 - .223]
Out of employment						
Full sample model	.198*** [.149 - .248]	-.027 [-.117 - .062]	-.131*** [-.176 - -.086]	-.080** [-.149 - -.010]	-.260*** [-.298 - -.222]	-.186*** [-.266 - -.106]
Males only	.252*** [.181 - .323]	-.029 [-.149 - .090]	-.147*** [-.215 - -.078]	-.093* [-.200 - .014]	-.305*** [-.366 - -.245]	-.238*** [-.357 - -.118]
Females only	.183*** [.128 - .238]	-.046 [-.150 - .058]	-.096*** [-.149 - -.043]	-.071 [-.160 - .019]	-.222*** [-.267 - -.177]	-.157*** [-.248 - -.066]
Lack of community engagement						

Full sample model	.014<br>[-.019 - .048]	.021<br>[-.041 - .083]	-.062***<br>[-.096 - -.028]	-.007<br>[-.072 - .057]	-.049***<br>[-.079 - -.019]	-.002<br>[-.066 - .062]
Males only	-.002<br>[-.047 - .042]	.028<br>[-.054 - .111]	-.074***<br>[-.117 - -.030]	-.032<br>[-.119 - .055]	-.051**<br>[-.093 -.010]	-.010<br>[-.097 - .077]
Females only	.024<br>[-.018 - .065]	.017<br>[-.068 - .102]	-.055**<br>[-.100 - -.009]	.013<br>[-.069 - .095]	-.049**<br>[-.087 - -.010]	.011<br>[-.069 - .092]

Note: *All models use multiply imputed data, adjusted for the full covariate set and are run once with a cohort dummy variable and again to include a cohort interaction term (*cohort). Mental health outcomes are standardised, and continuous covariates centred. Coefficients are reported for independent and cohort interaction effects. Male and female samples do not = total sample due to some missing data on the sex at birth variable. Additional models were run for each social isolation indicator controlling for all other indicators to assess the independent associations net of the other social isolation domains – results were consistent with the above models.*

*** $p<0.01$, ** $p<0.05$, * $p<0.1$

Table S7. *Linear multivariable regression models for each separate social isolation indicator to identify (1) independent effects of each social isolation indicator and (2) cohort effects in these independent effects on psychological distress, life satisfaction and self-rated general health – complete case*

All coefficients presented are for each independent social isolation indicator only (exposure)	Psychological distress coef [95% CI] *N*		Life satisfaction coef [95% CI] *N*		Self-rated general health coef [95% CI] *N*	
	(1)Independent	(2)*Cohort	(1)Independent	(2)*Cohort	(1)Independent	(2)*Cohort
Living alone						
Full sample model	.011 [-.040 - .062] *11,806*	.041 [-.060 - .142] *11,806*	-.177*** [-.231 - -.124] *12,114*	.035 [-.071 - .142] *12,114*	-.066** [-.118 - -.014] *12,160*	-.138*** [-.242 - -.035] *12,160*
Males only	-.002 [-.063 - .059] *5,822*	-.017 [-.140 - .106] *5,822*	-.175*** [-.243 - -.106] *5,972*	.055 [-.082 - .192] *5,972*	-.080** [-.148 - -.012] *6,000*	-.129* [-.265 - .007] *6,000*
Females only	.051 [-.037 - .139] *5,663*	.143 [-.033 - .320] *5,663*	-.188*** [-.277 - -.099] *5,794*	.000 [-.177 - .177] *5,794*	-.061 [-.145 - .023] *5,811*	-.116 [-.284 - .052] *5,811*
Lack of regular contact with friends and relatives outside of the household						
Full sample model	.029 [-.079 - .137] *11,256*	-.226* [-.467 - .015] *11,256*	-.211*** [-.323 - -.099] *11,562*	-.024 [-.279 - .230] 11,562	-.031 [-.140 - .078] *11,604*	-.182 [-.430 - .066] *11,604*
Males only	.075 [-.055 - .205] *5,531*	-.331** [-.609 - -.054] *5,531*	-.226*** [-.367 - -.085] *5,681*	-.062 [-.369 - .245] *5,681*	-.085 [-.226 - .055] *5,710*	-.094 [-.401 - .212] *5,710*
Females only	-.025 [-.211 - .160] *5,404*	-.089 [-.532 - .353] *5,404*	-.152 [-.338 - .035] *5,533*	-.014 [-.463 - .435] *5,533*	.065 [-.113 - .243] *5,545*	-.335 [-.763 - .094] *5,545*

Out of employment						
Full sample model	.238***<br>[.170 - .305]<br>*11,807*	.090<br>[-.048 - .227]<br>*11,807*	-.198***<br>[-.269 - -.127]<br>*12,114*	-.154**<br>[-.297 - -.010]<br>*12,114*	-.302***<br>[-.371 - -.234]<br>*12,160*	-.147**<br>[-.286 - -.009]<br>*12,160*
Males only	.290***<br>[.178 - .402]<br>*5,824*	.111<br>[-.127 - .348]<br>*5,824*	-.245***<br>[-.370 - -.120]<br>*5,974*	-.412***<br>[-.676 - -.148]<br>*5,974*	-.442***<br>[-.565 - -.320]<br>*6,002*	-.454***<br>[-.713 - -.195]<br>*6,002*
Females only	*.228****<br>*[.139 - .317]*<br>*5,662*	.034<br>[-.147 - .216]<br>*5,662*	-.159***<br>[-.249 - -.070]<br>*5,792*	-.066<br>[-.247 - .115]<br>*5,792*	-.236***<br>[-.320 - -.151]<br>*5,809*	-.010<br>[-.181 - .161]<br>*5,809*
Lack of community engagement						
Full sample model	.021<br>[-.011 - .054]<br>*11,288*	-.023<br>[-.089 - .043]<br>*11,288*	-.065***<br>[-.100 - -.030]<br>*11,364*	-.009<br>[-.080 - .062]<br>*11,364*	-.032*<br>[-.066 - .002]<br>*11,403*	.001<br>[-.067 - .069]<br>*11,403*
Males only	-.001<br>[-.044 - .042]<br>*5,565*	-.001<br>[-.090 - .087]<br>*5,565*	-.087***<br>[-.137 - -.038]<br>*5,597*	-.033<br>[-.133 - .067]<br>*5,597*	-.062**<br>[-.110 - -.013]<br>*5,622*	-.002<br>[-.101 - .097]<br>*5,622*
Females only	.044*<br>[-.006 - .094]<br>*5,440*	-.033<br>[-.135 - .068]<br>*5,440*	-.040<br>[-.091 - .011]<br>*5,481*	-.001<br>[-.104 - .102]<br>*5,481*	-.012<br>[-.060 - .037]<br>*5,495*	.031<br>[-.067 - .129]<br>*5,495*

Note: *All models use complete case data, adjusted for the full covariate set and are run once with a cohort dummy variable and again to include a cohort interaction term (*cohort). Coefficients are reported for independent and cohort interaction effects.*

**** p<0.01, ** p<0.05, * p<0.1*

Table S8. *Linear multivariable regression models for each separate social isolation indicator to identify (1) independent effects of each social isolation indicator and (2) cohort effects in these independent effects on psychological distress, life satisfaction and self-rated general health using impute and delete data*

All coefficients presented are for each independent social isolation indicator only (exposure)	Psychological distress<br>*Total sample N = 17,500*<br>*Males N = 8,185*<br>*Females N = 8,746*<br>coef [95% CI]		Life satisfaction<br>*Total sample N = 18,118*<br>*Males N = 8,486*<br>*Females N = 9,008*<br>coef [95% CI]		Self-rated general health<br>*Total sample N = 18,309*<br>*Males N = 8,600*<br>*Females N = 9,079*<br>coef [95% CI]	
	(1)Independent	(2)*Cohort	(1)Independent	(2)*Cohort	(1)Independent	(2)*Cohort
Living alone						
Full sample model	.031<br>[-.017 - .079]	.023<br>[-.068 - .115]	-.207***<br>[-.257 - -.157]	.063<br>[-.034 - .161]	-.052**<br>[-.099 - -.005]	-.139***<br>[-.230 - -.048]
Males only	.033<br>[-.024 - .091]	-.030<br>[-.142 - .081]	-.190***<br>[-.255 - -.126]	.088<br>[-.035 - .210]	-.066**<br>[-.127 - -.006]	-.138**<br>[-.257 - -.020]
Females only	.046<br>[-.033 - .124]	.091<br>[-.067 - .248]	-.240***<br>[-.325 - -.156]	.026<br>[-.140 - .191]	-.044<br>[-.120 - .031]	-.123*<br>[-.268 - .022]
Lack of regular contact with friends and relatives outside of the household						
Full sample model	.047<br>[-.050 - .145]	-.190*<br>[-.416 - .035]	-.205***<br>[-.302 - -.108]	.052<br>[-.195 - .298]	-.088*<br>[-.181 - .006]	-.070<br>[-.293 - .153]
Males only	.035<br>[-.089 - .158]	-.207<br>[-.468 - .053]	-.185***<br>[-.315 - -.054]	.004<br>[-.292 - .301]	-.086<br>[-.211 - .038]	-.061<br>[-.332 - .211]
Females only	.071<br>[-.080 - .222]	-.175<br>[-.574 - .225]	-.234***<br>[-.391 - -.077]	.096<br>[-.324 - .515]	-.073<br>[-.223 - .077]	-.100<br>[-.476 - .277]
Out of employment						
Full sample model	.198***<br>[.154 - .243]	-.002<br>[-.083 - .079]	-.131***<br>[-.176 - -.086]	-.089**<br>[-.173 - -.005]	-.265***<br>[-.306 - -.224]	-.192***<br>[-.269 - -.114]
Males only	.307***<br>[.229 - .386]	.003<br>[-.142 - .148]	-.215***<br>[-.299 - -.131]	-.088<br>[-.239 - .064]	-.412***<br>[-.486 - -.337]	-.283***<br>[-.422 - -.144]
Females only	.158***	-.037	-.087***	-.065	-.202***	-.139***

	[.103 - .214]	[-.140 - .066]	[-.142 - -.032]	[-.171 - .040]	[-.253 - -.152]	[-.238 - -.041]
Lack of community engagement						
Full sample model	.012<br>[-.019 - .043]	.014<br>[-.047 - .076]	-.061***<br>[-.093 - -.029]	-.005<br>[-.072 - .062]	-.046***<br>[-.077 - -.015]	.004<br>[-.059 - .066]
Males only	-.015<br>[-.057 - .028]	.037<br>[-.050 - .124]	-.079***<br>[-.125 - -.032]	-.050<br>[-.145 - .045]	-.049**<br>[-.093 - -.004]	-.013<br>[-.103 - .078]
Females only	.038<br>[-.008 - .083]	-.005<br>[-.092 - .082]	-.045*<br>[-.091 - .001]	.026<br>[-.068 - .120]	-.044**<br>[-.087 - -.002]	.026<br>[-.057 - .110]

Note: *All models use multiply imputed data but with cohort members deleted who had missing data on outcomes, adjusted for the full covariate set and are run once with a cohort dummy variable and again to include a cohort interaction term (*cohort). Coefficients are reported for independent and cohort interaction effects. Male and female samples do not = total sample due to missing data on the sex at birth variable.*

**** p<0.01, ** p<0.05, * p<0.1*

Figure S1 (a, b, c and d). *Model predicted marginal mean plots for significant (p<.10) social isolation by cohort interactions on mental health outcomes*

a

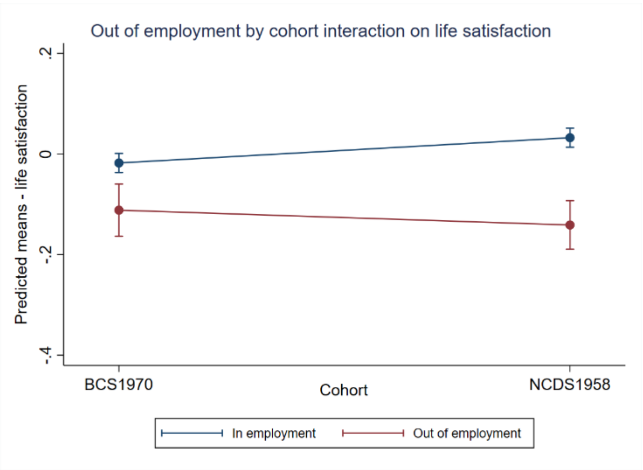

b

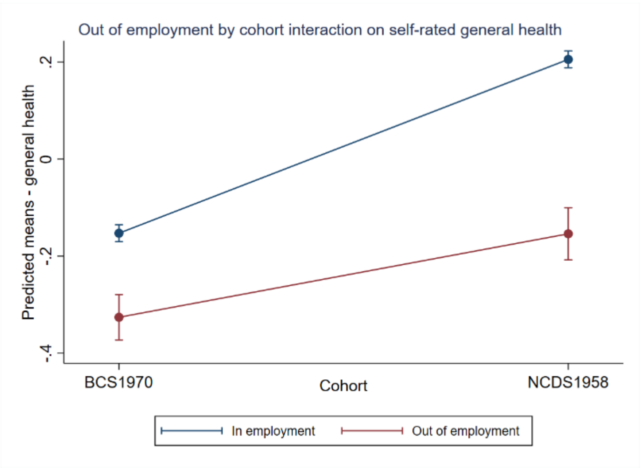

c

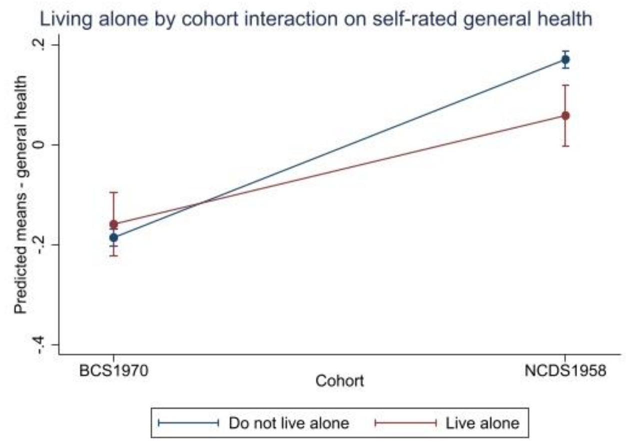

d

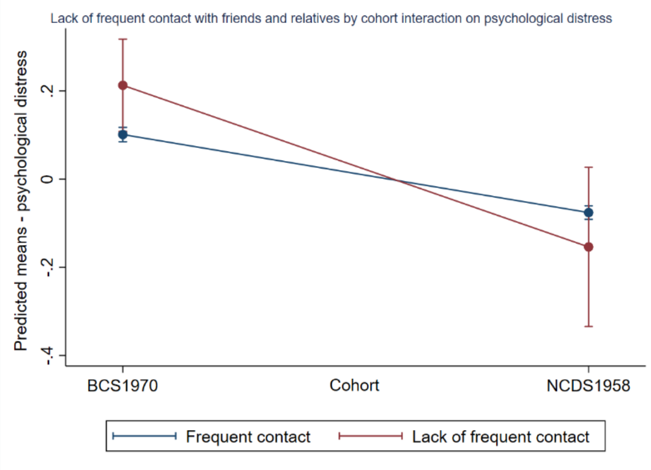

Figure S2. *Model predicted marginal mean plot for cumulative social isolation score by cohort interaction on self-rated general health*

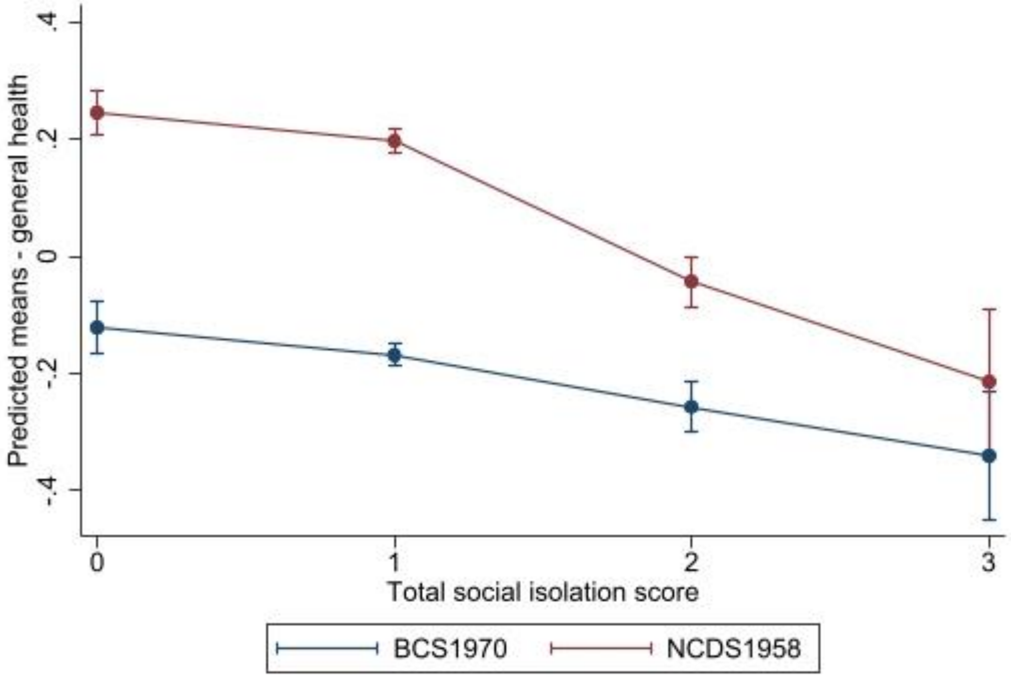

Table S9. *Linear multivariable regression models for the cumulative social isolation score to identify 1) cumulative effects using the cumulative social isolation score, and 2) cohort effects in any cumulative associations on psychological distress, life satisfaction and self-rated general health – complete case and impute and delete models*

All coefficients presented are for the cumulative social isolation score only (exposure)	Psychological distress coef [95% CI] *N*		Life satisfaction coef [95% CI] *N*		Self-rated general health coef [95% CI] *N*	
	(1)Cumulative effect	(2)*Cohort	(1)Cumulative effect	(2)*Cohort	(1)Cumulative effect	(2)*Cohort
Complete case						
Full sample model	.038***<br>[.012 - .064]<br>*10,411*	-.008<br>[-.059 - .043]<br>*10,411*	-.107***<br>[-.135 - -.080]<br>*10,487*	-.002<br>[-.056 - .052]<br>*10,487*	-.069***<br>[-.096 - -.042]<br>*10,520*	-.052*<br>[-.104 - .001]<br>*10,520*
Males only	.019<br>[-.015 - .052]<br>*5,083*	-.025<br>[-.092 - .042]<br>*5,083*	-.114***<br>[-.151 - -.076]<br>*5,115*	-.019<br>[-.094 - .056]<br>*5,115*	-.085***<br>[-.123 - -.048]<br>*5,140*	-.070*<br>[-.144 - .004]<br>*5,140*
Females only	.066***<br>[.026 - .106]<br>*5,045*	.010<br>[-.069 - .089]<br>*5,045*	-.094***<br>[-.134 - -.053]<br>*5,086*	-.002<br>[-.082 - .079]<br>*5,086*	-.060***<br>[-.099 - -.021]<br>*5,094*	-.014<br>[-.091 - .063]<br>*5,094*
Impute and delete						
Full sample model	.065***<br>[.044 - .086]<br>*17,500*	.003<br>[-.037 - .044]<br>*17,500*	-.116***<br>[-.138 - -.093]<br>*18,118*	-.004<br>[-.048 - .039]<br>*18,118*	-.105***<br>[-.126 - -.084]<br>*18,309*	-.077***<br>[-.117 - -.037]<br>*18,309*
Males only	.054***<br>[.025 - .083]<br>*8,185*	.002<br>[-.053 - .058]<br>*8,185*	-.132***<br>[-.165 - -.100]<br>*8,486*	-.006<br>[-.068 - .056]<br>*8,486*	-.114***<br>[-.144 - -.084]<br>*8,600*	-.091***<br>[-.149 - -.033]<br>*8,600*
Females only	.082***<br>[.051 - .114]<br>*8,746*	-.007<br>[-.066 - .053]<br>*8,746*	-.100***<br>[-.133- -.067]<br>*9,008*	.002<br>[-.061 - .066]<br>*9,008*	-.100***<br>[-.130 - -.071]<br>*9,079*	-.056**<br>[-.112 - .000]<br>*9,079*

Note: *All models are adjusted for the full covariate set and are run once with a cohort dummy variable and again to include a cohort interaction term (*cohort). Coefficients are reported for cumulative and cohort interaction effects.*

*** $p<0.01$, ** $p<0.05$, * $p<0.1$

Table S10. *Linear multivariable regression models for the interaction between multiple social isolation indicators to identify 1) the additive and multiplicative interaction effects, and 2) cohort effects in any interaction associations on psychological distress, life satisfaction and self-rated general health using multiply imputed data m=50 (Full sample N = 32,391, Males N = 15,810, Females N = 15,221)*

All coefficients presented are for interactions between multiple social isolation indicators (exposure)	Psychological distress coef [95% CI]		Life satisfaction coef [95% CI]		Self-rated general health coef [95% CI]	
	(1)Interaction	(2)*Cohort	(1)Interaction	(2)*Cohort	(1)Interaction	(2)*Cohort
Living alone Lack of regular contact with friends and relatives outside of the household*						
Full sample model	.114 [-.162 - .390]	-.212 [-.815 - .391]	-.038 [-.317 - .240]	-.099 [-.744 - .546]	.018 [-.236 - .273]	-.057 [-.673 - .559]
Males only	.098 [-.245 - .442]	-.164 [-.866 - .537]	.031 [-.313 - .375]	-.372 [-1.15 - .404]	-.054 [-.374 - .267]	-.123 [-.904 - .657]
Females only	.191 [-.328 - .709]	-.402 [-1.77 - .963]	-.172 [-.711 - .368]	.454 [-.874 - 1.78]	.115 [-.348 - .577]	.195 [-.938 - 1.33]
Living alone Out of employment*						
Full sample model	.077 [-.018 - .172]	-.111 [-.293 - .071]	-.097* [-.198 - .005]	.035 [-.160 - .231]	-.021 [-.114 - .070]	.052 [-.130 - .233]
Males only	.041 [-.089 - .171]	-.102 [-.352 - .148]	-.038 [-.175 - .099]	.033 [-.224 - .289]	.052 [-.074 - .178]	.079 [-.174 - .332]
Females only	.106 [-.058 - .270]	-.175 [-.472 - .123]	-.161* [-.337 - .015]	.052 [-.252 - .356]	-.078 [-.222 - .066]	.058 [-.228 - .345]
Living alone Lack of community engagement*						
Full sample model	-.010 [-.107 - .086]	-.003 [-.191 - .186]	.005 [-.103 - .113]	.001 [-.216 - .217]	.003 [-.099 - .104]	-.006 [-.192 - .180]
Males only	.029 [-.104 - .161]	.056 [-.199 - .311]	-.030 [-.179 - .118]	-.050 [-.341 - .240]	.004 [-.143 - .151]	-.079 [-.331 - .173]
Females only	-.053 [-.199 - .093]	-.084 [-.404 - .237]	.052 [-.109 - .213]	.051 [-.267 - .368]	.016 [-.137 - .170]	.078 [-.227 - .383]
*Lack of regular contact with friends and relatives outside of the household*Out of employment*						

Full sample model	.030<br>[-.149 - .209]	-.007<br>[-.455 - .441]	.020<br>[-.177 - .216]	.071<br>[-.410 - .551]	.052<br>[-.113 - .218]	.077<br>[-.370 - .524]
Males only	.014<br>[-.276 - .303]	.013<br>[-.539 - .566]	.049<br>[-.255 - .354]	.091<br>[-.612 - .794]	.110<br>[-.159 - .379]	.017<br>[-.572 - .606]
Females only	.055<br>[-.214 - .323]	-.092<br>[-.778 - .593]	-.002<br>[-.287 - .283]	.056<br>[-.731 - .844]	.037<br>[-.207 - .280]	.158<br>[-.543 - .858]
Lack of regular contact with friends and relatives outside of the household Lack of community engagement*						
Full sample model	.050<br>[-.191 - .29]	-.119<br>[-.771 - .534]	.086<br>[-.153 - .325]	-.215<br>[-.965 - .536]	-.045<br>[-.261 - .171]	.030<br>[-.656 - .717]
Males only	.098<br>[-.235 - .431]	-.116<br>[-.897 - .665]	.035<br>[-.324 - .395]	-.271<br>[-1.26 - .713]	-.046<br>[-.365 - .273]	.059<br>[-.802 - .919]
Females only	.016<br>[-.323 - .354]	-.240<br>[-1.39 - .909]	.130<br>[-.213 - .474]	-.057<br>[-1.26 - 1.15]	-.043<br>[-.362 - .275]	-.028<br>[-1.21 - 1.15]
Out of employment Lack of community engagement*						
Full sample model	.054<br>[-.024 - .132]	.056<br>[-.100 - .212]	-.035<br>[-.117 - .047]	.012<br>[-.154 - .179]	-.070*<br>[-.151 - .011]	-.000<br>[-.149 - .149]
Males only	.036<br>[-.112 - .184]	.055<br>[-.233 - .344]	-.071<br>[-.221 - .079]	-.088<br>[-.393 - .216]	-.003<br>[-.141 - .136]	.114<br>[-.143 - .372]
Females only	.052<br>[-.055 - .159]	.056<br>[-.143 - .254]	-.012<br>[-.126 - .102]	.057<br>[-.157 - .272]	-.096**<br>[-.192- -.000]	-.071<br>[-.268 - .126]

Note: *All models use multiply imputed data, adjusted for the full covariate set and are run once with a cohort dummy variable and again to include a cohort interaction term (*cohort). Mental health outcomes are standardised, and continuous covariates centred. Coefficients are reported for social isolation indicator interactions and cohort interaction effects. Male and female samples do not = total sample due to some missing data on the sex at birth variable.*

**** p<0.01, ** p<0.05, * p<0.1*

Table S11. *Linear multivariable regression models for the interaction between multiple social isolation indicators to identify 1) the additive and multiplicative interaction effects, and 2) cohort effects in any interaction associations on psychological distress, life satisfaction and self-rated general health using complete case data*

All coefficients presented are for interactions between multiple social isolation indicators (exposure)	Psychological distress<br>coef [95% CI]<br>p-value<br>*N*		Life satisfaction<br>coef [95% CI]<br>p-value<br>*N*		Self-rated general health<br>coef [95% CI]<br>p-value<br>*N*	
	(1)Interaction	(2)*Cohort	(1)Interaction	(2)*Cohort	(1)Interaction	(2)*Cohort
Living alone Lack of regular contact with friends and relatives outside of the household*						
Full sample model	.178<br>[-.289 - .644]<br>*10,929*	-.537<br>[-1.59 - .513]<br>*10,929*	-.030<br>[-.524 - .464]<br>*11,236*	-.593<br>[-1.71 - .520]<br>*11,236*	.291<br>[-.191 - .774]<br>*11,276*	-.447<br>[-1.53 - .640]<br>*11,276*
Males only	-.068<br>[-.698 - .562]<br>*5,340*	-.156<br>[-1.45 - 1.14]<br>*5,340*	-.175<br>[-.878 - .529]<br>*5,490*	-1.54**<br>[-2.99 - -.097]<br>*5,490*	.094<br>[-.609 - .796]<br>*5,518*	-.895<br>[-2.34 - .549]<br>*5,518*
Females only	.524<br>[-.302 - 1.35]<br>*5,268*	-1.33<br>[-3.25 - .590]<br>*5,268*	.140<br>[-.699 - .978]<br>*5,398*	1.02<br>[-.923 - 2.97]<br>*5,398*	.825**<br>[.022 - 1.63]<br>*5,409*	.080<br>[-1.78 - 1.94]<br>*5,409*
Living alone Out of employment*						
Full sample model	.046<br>[-.174 - .266]<br>*11,802*	-.206<br>[-.676 - .265]<br>*11,802*	-.128<br>[-.362 - .107]<br>*12,109*	.231<br>[-.270 - .731]<br>*12,109*	-.231***<br>[-.455 - -.007]<br>*12,155*	.319<br>[-.163 - .801]<br>*12,155*
Males only	.013<br>[-.274 - .300]<br>*5,820*	-.399<br>[-1.26 - .477]<br>*5,820*	-.099<br>[-.423 - .225]<br>*5,970*	1.43***<br>[.447 - 2.42]<br>*5,970*	-.097<br>[-.410 - .216]<br>*5,998*	-.057<br>[-1.04 - .920]<br>*5,998*
Females only	.041<br>[-.309 - .391]<br>*5,661*	-.145<br>[-.850 - .559]<br>*5,661*	-.143<br>[-.499 - .213]<br>*5,791*	-.264<br>[-.980 - .453]<br>*5,791*	-.279<br>[-.617 - .059]<br>*5,808*	.652*<br>[-.028 - 1.33]<br>*5,808*
Living alone Lack of community engagement*						

Full sample model	-.082<br>[-.210 - .047]<br>*11,283*	.036<br>[-.225 - .298]<br>*11,283*	.036<br>[-.102 - .174]<br>*11,359*	-.009<br>[-.290 - .271]<br>*11,359*	.006<br>[-.127 - .139]<br>*11,398*	-.075<br>[-.346 - .196]<br>*11,398*
Males only	.018<br>[-.148 - .184]<br>*5,561*	.187<br>[-.150 - .523]<br>*5,561*	-.036<br>[-.226 - .153]<br>*5,593*	-.173<br>[-.557 - .211]<br>*5,593*	-.023<br>[-.210 - .165]<br>*5,618*	-.383**<br>[-.763 - -.003]<br>*5,618*
Females only	-.154<br>[-.358 - .051]<br>*5,439*	-.129<br>[-.555 - .297]<br>*5,439*	.122<br>[-.087 - .330]<br>*5,480*	.109<br>[-.326 - .543]<br>*5,480*	.087<br>[-.111 - .285]<br>*5,494*	.192<br>[-.221 - .605]<br>*5,494*
*Lack of regular contact with friends and relatives outside of the household*Out of employment*						
Full sample model	-.271<br>[-.739 - .197]<br>*10,931*	.509<br>[-.694 - 1.71]<br>*10,931*	.138<br>[-.359 - .635]<br>*11,238*	.123<br>[-1.15 - 1.40]<br>*11,238*	.473*<br>[-.011 - .956]<br>*11,278*	-.854<br>[-2.10 - .389]<br>*11,278*
Males only	.211<br>[-.496 - .917]<br>*5,343*	-.354<br>[-1.78 - 1.07]<br>*5,343*	.269<br>[-.523 - 1.06]<br>*5,493*	.180<br>[-1.41 - 1.77]<br>*5,493*	.410<br>[-.377 - 1.20]<br>*5,521*	-.915<br>[-2.50 - .669]<br>*5,521*
Females only	-.320<br>[-1.01 - .366]<br>*5,267*	-	.159<br>[-.539 - .857]<br>*5,397*	-	.547<br>[-.118 - 1.21]<br>*5,408*	-
Lack of regular contact with friends and relatives outside of the household Lack of community engagement*						
Full sample model	.197<br>[-.137 - .531]<br>*10,419*	-.627<br>[-1.76 - .505]<br>*10,419*	.312*<br>[-.043 - .667]<br>*10,495*	.097<br>[-1.11 - 1.30]<br>*10,495*	-.214<br>[-.559 - .132]<br>*10,528*	.713<br>[-.461 - 1.89]<br>*10,528*
Males only	.319<br>[-.100 - .739]<br>*5,088*	-.446<br>[-1.90 - 1.00]<br>*5,088*	.310<br>[-.163 - .782]<br>*5,120*	.079<br>[-1.56 - 1.72]<br>*5,120*	-.130<br>[-.599 - .340]<br>*5,145*	.938<br>[-.690 - 2.57]<br>*5,145*
Females only	.078<br>[-.450 - .606]<br>*5,048*	-.846<br>[-2.59 - .901]<br>*5,048*	.355<br>[-.181 - .891]<br>*5,089*	-.026<br>[-1.80 - 1.75]<br>*5,089*	-.284<br>[-.797 - .230]<br>*5,097*	.420<br>[-1.28 - 2.12]<br>*5,097*

Out of employment Lack of community engagement*						
Full sample model	.197**<br>[.042 - .353]<br>*11,285*	-.096<br>[-.417 - .225]<br>*11,285*	-.179**<br>[-.346 - -.012]<br>*11,361*	.002<br>[-.342 - .345]<br>*11,361*	-.206**<br>[-.368 - -.044]<br>*11,400*	-.209<br>[-.542 - .124]<br>*11,400*
Males only	.186<br>[-.104 - .475]<br>*5,564*	-.526*<br>[-1.13 - .075]<br>*5,564*	-.381**<br>[-.711- -.051]<br>*5,596*	.139<br>[-.547 - .824]<br>*5,596*	-.129<br>[-.455 - .196]<br>*5,621*	.421<br>[-.256 - 1.10]<br>*5,621*
Females only	.199**<br>[.000 - .398]<br>*5,438*	.003<br>[-.409 - .414]<br>*5,438*	-.127<br>[-.330 - .076]<br>*5,479*	-.023<br>[-.443 - .397]<br>*5,479*	-.218**<br>[-.411 - -.025]<br>*5,493*	-.400**<br>[-.799 - -.001]<br>*5,493*

Note: *All models use complete case data, adjusted for the full covariate set and are run once with a cohort dummy variable and where significant, again to include a cohort interaction term (*cohort) and for males and females only. Mental health outcomes are standardised, and continuous covariates centred. Coefficients are reported for social isolation indicator interactions and cohort interaction effects. Male and female samples do not = total sample due to some missing data on the sex at birth variable. - = no observations in the sample.*

**** p<0.01, ** p<0.05, * p<0.1*

Table S12. *Linear multivariable regression models for the interaction between multiple social isolation indicators to identify 1) the additive and multiplicative interaction effects, and 2) cohort effects in any interaction associations on psychological distress, life satisfaction and self-rated general health using impute and delete data*

All coefficients presented are for interactions between multiple social isolation indicators (exposure)	Psychological distress<br>*Total sample N = 17,500*<br>*Males N = 8,185*<br>*Females N = 8,746*<br>coef [95% CI]<br>p-value		Life satisfaction<br>*Total sample N = 18,118*<br>*Males N = 8,486*<br>*Females N = 9,008*<br>coef [95% CI]<br>p-value		Self-rated general health<br>*Total sample N = 18,309*<br>*Males N = 8,600*<br>*Females N = 9,079*<br>coef [95% CI]<br>p-value	
	(1)Interaction	(2)*Cohort	(1)Interaction	(2)*Cohort	(1)Interaction	(2)*Cohort
Living alone Lack of regular contact with friends and relatives outside of the household*						
Full sample model	.238<br>[-.123 - .600]	-.490<br>[-1.33 - .351]	-.044<br>[-.409 - .320]	-.231<br>[-1.13 - .666]	.064<br>[-.269 - .397]	-.197<br>[-1.07 - .672]
Males only	.154<br>[-.320 - .628]	-.336<br>[-1.35 - .675]	.145<br>[-.351 - .642]	-.907<br>[-2.08 - .262]	-.050<br>[-.525 - .425]	-.371<br>[-1.44 - .693]
Females only	.357<br>[-.298 - 1.01]	-.848<br>[-2.62 - .923]	-.359<br>[-1.06 - .338]	.985<br>[-.930 - 2.90]	.211<br>[-.332 - .755]	.365<br>[-1.25 - 1.98]
Living alone Out of employment*						
Full sample model	.165**<br>[.033 - .297]	-.288**<br>[-.544 - -.032]	-.197***<br>[-.335 - -.058]	.131<br>[-.146 - .408]	-.049<br>[-.169 - .072]	.123<br>[-.114 - .359]
Males only	.082<br>[-.080 - .244]	-.311*<br>[-.641 - .019]	-.024<br>[-.206 - .157]	.107<br>[-.272 - .486]	.137<br>[-.027 - .300]	.146<br>[-.178 - .471]
Females only	.223**<br>[.009 - .437]	-.406*<br>[-.826 - .014]	-.333***<br>[-.556 - -.111]	.137<br>[-.309 - .583]	-.137<br>[-.324 - .051]	.174<br>[-.196 - .544]
Living alone Lack of community engagement*						
Full sample model	-.022<br>[-.140 - .095]	.023<br>[-.218 - .264]	-.008<br>[-.130 - .113]	.016<br>[-.244 - .277]	.013<br>[-.105 - .130]	-.041<br>[-.275 - .194]
Males only	.058<br>[-.096 - .213]	.104<br>[-.223 - .431]	-.081<br>[-.251 - .089]	-.066<br>[-.421 - .288]	.004<br>[-.157 - .165]	-.174<br>[-.504 - .157]

Females only	-.084 [-.269 - .100]	-.088 [-.473 - .297]	.078 [-.115 - .271]	.070 [-.323 - .462]	.055 [-.127 - .238]	.104 [-.259 - .466]
*Lack of regular contact with friends and relatives outside of the household*Out of employment*						
Full sample model	.024 [-.251 - .299]	.047 [-.603 - .697]	.061 [-.221 - .343]	.114 [-.544 - .773]	.094 [-.146 - .333]	.055 [-.550 - .660]
Males only	-.007 [-.432 - .419]	.146 [-.716 - 1.01]	.163 [-.329 - .656]	.154 [-.841 - 1.15]	.300 [-.104 - .703]	-.102 [-.925 - .722]
Females only	.042 [-.358 - .442]	-.156 [-1.20 - .888]	-.017 [-.426 - .391]	.107 [-.938 - 1.15]	.015 [-.326 - .355]	.200 [-.763 - 1.16]
Lack of regular contact with friends and relatives outside of the household Lack of community engagement*						
Full sample model	.127 [-.158 - .413]	-.253 [-1.07 - .561]	.133 [-.174 - .440]	-.312 [-1.21 - .588]	-.056 [-.333 - .221]	.134 [-.708 - .977]
Males only	.232 [-.153 - .618]	-.308 [-1.28 - .666]	.048 [-.370 - .467]	-.413 [-1.53 - .708]	-.060 [-.449 - .328]	.186 [-.853 - 1.23]
Females only	.057 [-.358 - .473]	-.488 [-2.05 - 1.08]	.195 [-.250 - .641]	.075 [-1.55 - 1.70]	-.044 [-.472 - .385]	.038 [-1.51 - 1.59]
Out of employment Lack of community engagement*						
Full sample model	.109** [.010 - .208]	.060 [-.122 - .242]	-.037 [-.140 - .067]	.027 [-.166 - .221	-.127*** [-.217 - -.036]	.042 [-.142 - .225]
Males only	.103 [-.087 - .294]	.101 [-.269 - .470]	-.140 [.358 - .078]	-.226 [-.639 - .187]	.013 [-.183 - .209]	.282 [-.103 - .666]
Females only	.079 [-.042 - .201]	.051 [-.182 - .284]	.005 [-.121 - .131]	.100 [-.144 - .344]	-.144*** [-.251 - -.036]	-.059 [-.286 - .168]

Note: *All models use impute and delete data, adjusted for the full covariate set and are run once with a cohort dummy variable and where significant, again to include a cohort interaction term (*cohort) and for males and females only. Mental health outcomes are standardised, and continuous covariates centred. Coefficients are reported for social isolation indicator interactions and cohort interaction effects. Male and female samples do not = total sample due to some missing data on the sex at birth variable.*

**** p<0.01, ** p<0.05, * p<0.1*

Latent Class Analysis Methodology

Latent class analysis was adopted to identify groups of individuals showing qualitatively similar patterns in their social conditions (Weller et al., 2020). First, latent class analysis generates probabilities for who belongs to which group or class. Then, the models describe the relationship between classes and the observed variables. To understand the optimal number of social isolation groups within the current data, models were run estimating an increasing number of classes between 1 and 5. When selecting the class solution, goodness-of-fit statistics were used in combination with a theoretical interpretation (Weller et al., 2020). The Bayesian information criteria (BIC) is the most commonly used criteria, with a lower BIC indicating better fit (Killian et al., 2019). We assessed both the BIC and the adjusted Bayesian information criteria (ABIC). The Akaike information criterion (AIC) indicated the adequacy of different non-nested models, where lower IC indicates a more adequate model (Wagenmakers & Farrell, 2004). In addition to these ICs, we calculated the entropy and posterior class-membership probabilities (Celeux & Soromenho, 1996) where an entropy value closer to 1 is preferable, and average latent posterior probabilities of greater than .90 are desirable (Muthén & Muthén, 2000). The Vuong-Lo-Mendell-Rubin adjusted likelihood ratio test also helped determine the final class solution (Lo et al., 2001; Vuong et al., 1989). Finally, we considered the general rule that classes should not contain less than 5% of the sample (Shanahan et al., 2013). Latent classes were assessed by sex and cohort. A Full Information Maximum Likelihood (FIML) approach to handling missing data was applied in Mplus eighth edition (Muthén & Muthén, 2017) for the latent class analyses.

Table S13. *Results from the latent class analyses (1-5 classes) including goodness-of-fit statistics (full sample with 1970 BCS and 1958 cohorts combined N=19,902)*

Class solution	BIC	ABIC	AIC	Entropy	Average latent posterior probabilities (>.90) yes/no	Vuong-Lo-Mendell-Rubin adjusted likelihood ratio test (p-value)	Any classes with less than 5% of the sample yes/no
1	48779.359	48766.647	48747.764	-	-	-	-
2	48719.095	48690.493	48648.008	.985	yes	.000	no
3	48732.183	48687.692	48621.603	.932	no	.000	yes
4	48776.514	48716.133	48626.441	.836	no	.108	yes
5	48823.570	48747.299	48634.004	.462	no	.732	yes

Note: *BIC = Bayesian information criteria, ABIC = adjusted Bayesian information criteria, AIC = Akaike information criterion. Sample based on FIML approach in Mplus*

Given similar BIC, ABIC, AIC and entropy values when compared to the two-class solution, and a significant Vuong-Lo-Mendell-Rubin adjusted likelihood ratio test indicating a better fit to the data, the three-class solution was identified as the optimal class solution for these data. Despite all groups in the three-class solution being characterised by a high probability of limited community engagement, the first class included those who also lived alone, the second class those who lacked regular contact with friends and relatives outside the household, and the third class had a low probability of isolation across all contexts except the community.

Table S14. *Class membership (N(%)) for full sample and by sex and cohort*

Class	Full sample *N=19,902*	Males *N= 8,371*	Females *N=9,083*	1970 BCS *N=9,802*	1958 NCDS *N=10,100*
1 – living alone and lacking community engagement	*393*(1.97)	*156*(1.86)	*111*(1.22)	*223*(2.28)	*170*(1.68)
2 – lack of frequent contact with friends and relatives outside the household and lacking community engagement	*392*(1.97)	*170*(2.03)	*124*(1.37)	*317*(3.23)	*75*(0.74)
3 – low isolation except lack of community engagement	19,117(96.06)	*8,045*(96.11)	*8,848*(97.41)	*9,262*(94.49)	*9,855*(97.57)

Note: *sample based on FIML approach in Mplus and complete case sex*
